# Multi-faceted computational assessment of risk and progression in oligodendroglioma implicates NOTCH and PI3K pathways

**DOI:** 10.1038/s41698-018-0067-9

**Published:** 2018-11-06

**Authors:** Sameer H. Halani, Safoora Yousefi, Jose Velazquez Vega, Michael R. Rossi, Zheng Zhao, Fatemeh Amrollahi, Chad A. Holder, Amelia Baxter-Stoltzfus, Jennifer Eschbacher, Brent Griffith, Jeffrey J. Olson, Tao Jiang, Joseph R. Yates, Charles G. Eberhart, Laila M. Poisson, Lee A. D. Cooper, Daniel J. Brat

**Affiliations:** 1 Emory University School of Medicine, Atlanta, GA USA; 2 Department of Biomedical Informatics, Emory University, Atlanta, GA USA; 3 Department of Pathology and Laboratory Medicine, Emory University, Atlanta, GA USA; 4 Department of Neurosurgery, Tiantan Hospital, Capital Medical University, Beijing, China; 5 Department of Radiology, Emory University, Atlanta, GA USA; 6 Department of Neuropathology, Barrow Neurological Institute, Phoenix, AZ USA; 7 Department of Radiology, Henry Ford Health System, Detroit, MI USA; 8 Josephine Ford Cancer Institute, Henry Ford Health System, Detroit, MI USA; 9 Department of Neurosurgery, Emory University, Atlanta, GA USA; 10 Winship Cancer Institute, Emory University, Atlanta, GA USA; 11 Divisions of Pathology, Ophthalmology, and Oncology, Johns Hopkins University School of Medicine, Baltimore, MD USA; 12 Department of Public Health Sciences, Henry Ford Hospital Systems, Detroit, MI USA; 13 Department of Biomedical Engineering, Emory University/Georgia Institute of Technology, Atlanta, GA USA; 14 Department of Pathology, Northwestern University Feinberg School of Medicine, Chicago, IL USA

## Abstract

Oligodendrogliomas are diffusely infiltrative gliomas defined by *IDH*-mutation and co-deletion of 1p/19q. They have highly variable clinical courses, with survivals ranging from 6 months to over 20 years, but little is known regarding the pathways involved with their progression or optimal markers for stratifying risk. We utilized machine-learning approaches with genomic data from The Cancer Genome Atlas to objectively identify molecular factors associated with clinical outcomes of oligodendroglioma and extended these findings to study signaling pathways implicated in oncogenesis and clinical endpoints associated with glioma progression. Our multi-faceted computational approach uncovered key genetic alterations associated with disease progression and shorter survival in oligodendroglioma and specifically identified Notch pathway inactivation and PI3K pathway activation as the most strongly associated with MRI and pathology findings of advanced disease and poor clinical outcome. Our findings that Notch pathway inactivation and PI3K pathway activation are associated with advanced disease and survival risk will pave the way for clinically relevant markers of disease progression and therapeutic targets to improve clinical outcomes. Furthermore, our approach demonstrates the strength of machine learning and computational methods for identifying genetic events critical to disease progression in the era of big data and precision medicine.

## Brain cancer: AI reveals pathways implicated in oligodendroglioma

Using artificial intelligence, researchers identified two key signaling pathways involved in the progression of a brain cancer known as oligodendroglioma. A team led by Daniel Brat from Northwestern University Feinberg School of Medicine in Chicago, Illinois, and Lee Cooper from Emory University in Atlanta, Georgia, took a machine-learning approach to find links between genomic records, radiographic brain imaging data and digitized pathology slides from patients with oligodendroglioma, a type of tumor that develops from brain cells known as an oligodendrocytes. Their computational model identified an association between worse clinical outcomes and genetic alterations that either inactivate the Notch signaling pathway or activate the phosphoinositide 3-kinase pathway. The findings could yield potential therapeutic targets or prognostic biomarkers, although more work is needed to elucidate the exact proteins in these two pathways that drive disease progression.

## Introduction

Oligodendrogliomas are diffuse gliomas characterized by *IDH*-mutation, co-deletion of 1p/19q and *TERT* promoter mutations. They have the least aggressive clinical course among this group, yet display widely variable outcomes—some patients survive 6 months while others live over 20 years.^1–5^ Aside from their defining genetic alterations, oligodendrogliomas also harbor other mutations, including: capicua transcriptional repressor (*CIC*) (62%), far upstream element binding protein 1 (*FUBP1)* (27–29%)*, NOTCH1* (18–31%), catalytic and regulatory subunits of phosphoinositide-3-kinase (PI3K*; PIK3CA* (15–20%) and *PIK3R1* (7–9%), respectively), and others.^1,6,7^ Now that lower-grade gliomas are understood in objective, molecular terms, markers of progression and targets of therapy are being evaluated in a pure cohort, without the confounding contamination of dissimilar tumor types. Recent investigations by Aoki et al.^8^ for example, indicated that NOTCH1 mutations were associated with poor clinical outcomes in patients with oligodendroglioma.

With the tremendous expansion of genomic data available for both investigation and potential clinical care, a need has developed for novel computational approaches to investigate risk factors in a highly multidimensional and interdependent space.^9^ Machine-learning approaches are capable of using large genomic datasets in a manner that adds value to traditional risk modeling by identifying key prognostic factors among tens of thousands of possible variables. We employed machine-learning to identify molecular factors associated with clinical outcomes of oligodendroglioma using The Cancer Genome Atlas (TCGA) LGG dataset. We advanced and translated these findings using neuroimaging and pathology imaging features of progression to identify molecular biomarkers most closely related to advanced disease status, as defined by: (1) contrast-enhancement on magnetic resonance imaging (MRI); (2) high cellular density in digitized histopathologic images; and (3) increased cellular proliferation.^10–12^ In addition, our approach enabled us to identify key signaling pathways associated with more aggressive disease in addition to individual biomarkers. Our approach confirmed the association of NOTCH1 mutations with disease progression and shorter survival in oligodendroglioma, and further uncovered aberrant regulation of Notch and PI3K pathways as most strongly associated with advanced disease.

## Results

### Patient and tumor characteristics

The clinical factors from the 169 oligodendroglioma patients included in our study are presented in Table 1. *TERT* promoter mutations were present in 98% (86 of 88).^13^

**Table 1:** Patient demographics

Characteristic	Total (*N* = 169)
Original histologic diagnosis (WHO 2007)—no. (%)	
Oligodendroglioma	
Grade II	62 (36.7)
Grade III	55 (32.5)
Oligoastrocytoma	
Grade II	17 (10.1)
Grade III	13 (7.7)
Astrocytoma	
Grade II	2 (1.2)
Grade III	2 (1.2)
Age at diagnosis (yrs)	
Mean ± SD	45.8 ± 12.8
Range	17–75
Male sex—no. (%)	84 (49.7)
White race—no./total no. (%)	155/164 (94.5)
Extent of resection—no./total no. (%)	
Open biopsy	1/164 (0.6)
Subtotal resection	59/164 (36.0)
Gross total resection	104/164 (63.4)
Tumor location—no./total no. (%)	
Frontal lobe	122/166 (73.5)
Occipital lobe	3/166 (1.8)
Parietal lobe	14/166 (8.4)
Temporal lobe	27/166 (16.3)
Laterality—no/total no. (%)	
Left	79/168 (47.0)
Midline	3/168 (1.8)
Right	86/168 (51.2)

Clinical characteristics of patients from The Cancer Genome Atlas database with confirmed diagnosis of oligodendroglioma (i.e., *IDH*-mutant, 1p19q co-deleted glioma).

### Neural network analyses identifies molecular factors associated with outcomes

**Fig. 1:**
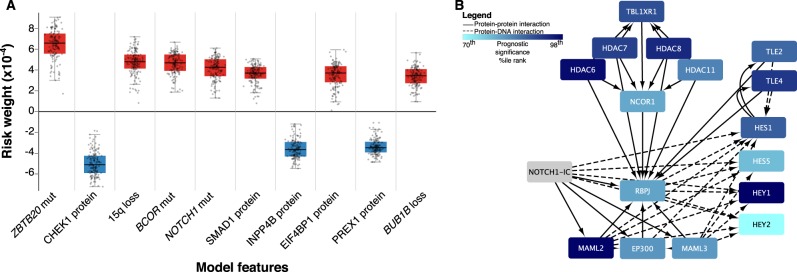
**a** Neural network risk factors. A nonlinear Cox proportional hazards model was trained using a neural network to model survival in oligodendrogliomas using clinical, genetic and proteomic factors. Prognostic significance of each feature was assessed by determining how its changes impact prognosis. Positive scores indicate a negative impact on survival (red) while negative scores (blue) suggest a positive impact. The boxplot contains the top 10 factors ranked by median prognostic importance; complete results in Datafile S1. **b** Gene set enrichment analysis of Notch pathway members. A separate model based on mRNA expression weighed the prognostic significance of individual transcripts and used this data in a gene-set-enrichment analysis to identify pathways associated with prognosis. The canonical Notch pathway was highly enriched with significantly negatively scored transcripts (i.e., darker blue signifies negative scores). Increased expression of downstream targets, including *HES1*, *HES5*, and *HEY1*, were associated with improved prognosis. This model demonstrates Notch signaling inactivation is associated with poor prognosis

Analysis of the genetic-protein neural network model revealed multiple mutations, CNAs, and proteins associated with overall survival in oligodendrogliomas (see Fig. 1a). *NOTCH1* (rank #5), *BCOR* (rank #4), and *ZBTB20* (rank #1) mutations were among the most highly ranked factors associated with poor prognosis, along with loss of 15q (rank #3). Both *NOTCH1* mutations and 15q loss occur in a substantial subset of oligodendrogliomas and have previously been suggested as markers of poor prognosis in traditional risk models,^14^ providing support for our model. The complete list of ranked factors is in the Supplementary Materials (Data file S1). Among these factors, we focused on the Notch pathway since *NOTCH1* mutations are relatively specific to oligodendroglioma among diffuse gliomas; occur in a substantial subset (18–31%) compared to *BCOR* and *ZBTB20*; and represent one component of the Notch signaling network that could be more generally relevant to disease progression. PI3K pathway subunit mutations were also of interest since they were heavily enriched among highly ranked negative prognostic factors (*PIK3R1*, #30; *PIK3CA*, #193).

Similar analysis of the gene expression neural network model was performed to determine the prognostic importance of mRNA transcripts, and a gene-set-enrichment analysis (GSEA) was then used to identify molecular pathways enriched with prognostic transcripts. GSEA identified the NOTCH1 Intracellular Domain Regulates Transcription pathway (*P* = 0.004) as highly enriched in transcripts associated with better prognosis, suggesting that Notch pathway inactivation is associated with poor outcomes (Fig. 1b). Regulation of KIT Signaling was also significantly enriched with positive prognosis transcripts (*P* = 0.002). The P38 / MKK3 (*P* < 0.05) and SMAD2 / SMAD3 pathways (*P* = 0.002) were also significantly enriched in transcripts associated with a poor prognosis, and notably, both interface directly with the PI3K pathway.^15,16^

The results of Monte-Carlo cross validation of the genetic-protein and gene expression survival neural networks are presented in Supplementary Figure S1. The median c-index of the tested genetic-protein models was 0.8 (±0.124), while the median c-index of the tested gene expression models was 0.752 (±0.196).

### Radiographic and pathologic features are associated with aggressive clinical behavior

We next focused on mutations and CNAs with a > 5% incidence to assess their association with radiographic and pathologic measures of disease progression, including: mutations of *CIC* (ranked #107; 61.5% incidence) *NOTCH1* (ranked #5; 18.9%), *FUBP1* (ranked #20; 27.2%), both *PIK3* subunits (*PIK3R1* ranked #30 and *PIK3CA* ranked #193; 23.1%), and CNA’s including gain of chromosomal arms 7p (ranked #300; 8.9%) and 11p (ranked #153; 11.2%), as well as loss of 14q (ranked #310; 11.8%) and 15q (ranked #3; 16.6%) (Fig. S2 illustrates a waterfall plot of the most frequent genetic alterations; Table S1**)**.

**Fig. 2:**
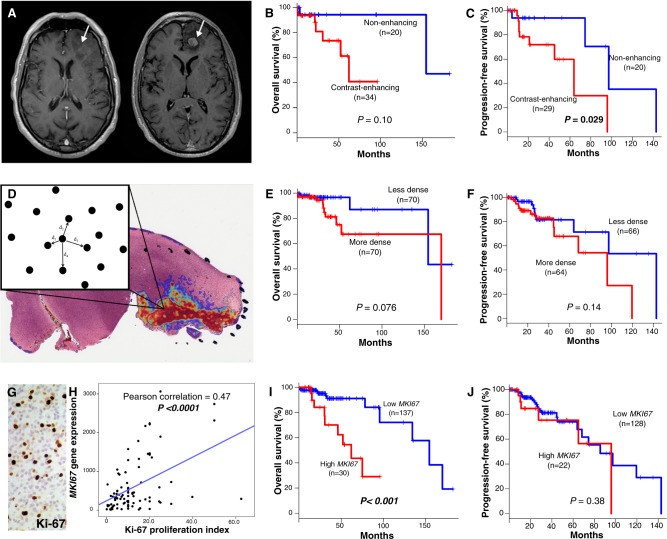
Markers of disease progression in oligodendroglioma **a** T1-weighted axial MR images with gadolinium contrast demonstrating CE− (left) and CE+ (right) features of oligodendroglioma from The Cancer Imaging Archive. **b** Kaplan–Meier plots of overall survival (OS) for CE- vs. CE + . **c** Progression-free survival (PFS) for CE− vs. CE+. **d** Visual representation of a tumor heatmap showing regions of interest of cell density, with a schematic diagram of the nearest-neighbor algorithm. **e** OS for cellular density (less vs. more dense). **f** PFS for less vs. more dense. **g**. High Ki-67 proliferation index visualized with IHC. **h** Linear regression of *MKI67* expression and Ki-67 proliferation index approximated by IHC. **i** OS for high vs. low *MKI67*. **j** PFS for high vs. low *MKI67*. *P* values for survival plots determined using log-rank tests

Contrast-enhancement observed on MRI is a well-known marker of higher-grade disease (Fig. 2a). Among 55 patients with MRI images available, contrast-enhancing (CE+) tumors (*n* = 35) had worse overall survival (OS) (median, 154.3 vs. 62.0 months; *P* = 0.10) and progression-free survival (PFS) (median, 97.3 vs. 63.8 months; *P* *=* 0.029) compared to those lacking enhancement (CE−) (*n* = 20) (Figs. 2b, c). CE+ was highly enriched for histologic grade III tumors; 24 of 25 grade III tumors were CE+ (*P* *<* 0.0001).

Since cell density increases with disease progression, we used a computational nearest-neighbor analysis to quantify cellular density in tissue sections from 142 cases (Fig. 2d). Higher cell density trended towards worse OS (mean 152.8 vs. 126.1 months; *P* = 0.076) and worse PFS (median 142.8 vs. 95.9 months; *P* = 0.14) (Figs. 2e, f). High cell density cases were also enriched for histologic grade III tumors; 44 of 58 high density tumors were WHO grade III (*P* *<* 0.0001).

As a measure of proliferation, *MKI67* mRNA expression was analyzed for 169 tumors. *MKI67* expression was strongly correlated with Ki-67/MIB-1 proliferation indices based on immunohistochemistry (IHC) and listed in TCGA pathology reports (*P* < 0.0001) (Figs. 2g, h). Patients with high cellular proliferation (*n* = 31) had worse OS (median 154.3 vs. 62.0 months; *P* = 0.001); no significant difference was noted in PFS (*P* = 0.38) (Figs. 2i, j). Highly proliferative tumors were also enriched for histologic grade III tumors; 21 of 28 high proliferation tumors with grade information available were WHO grade III (*P* *=* 0.001).

### Genetic alterations associated with radiographic contrast enhancement, cellular density, and MKI67 expression

**Fig. 3:**
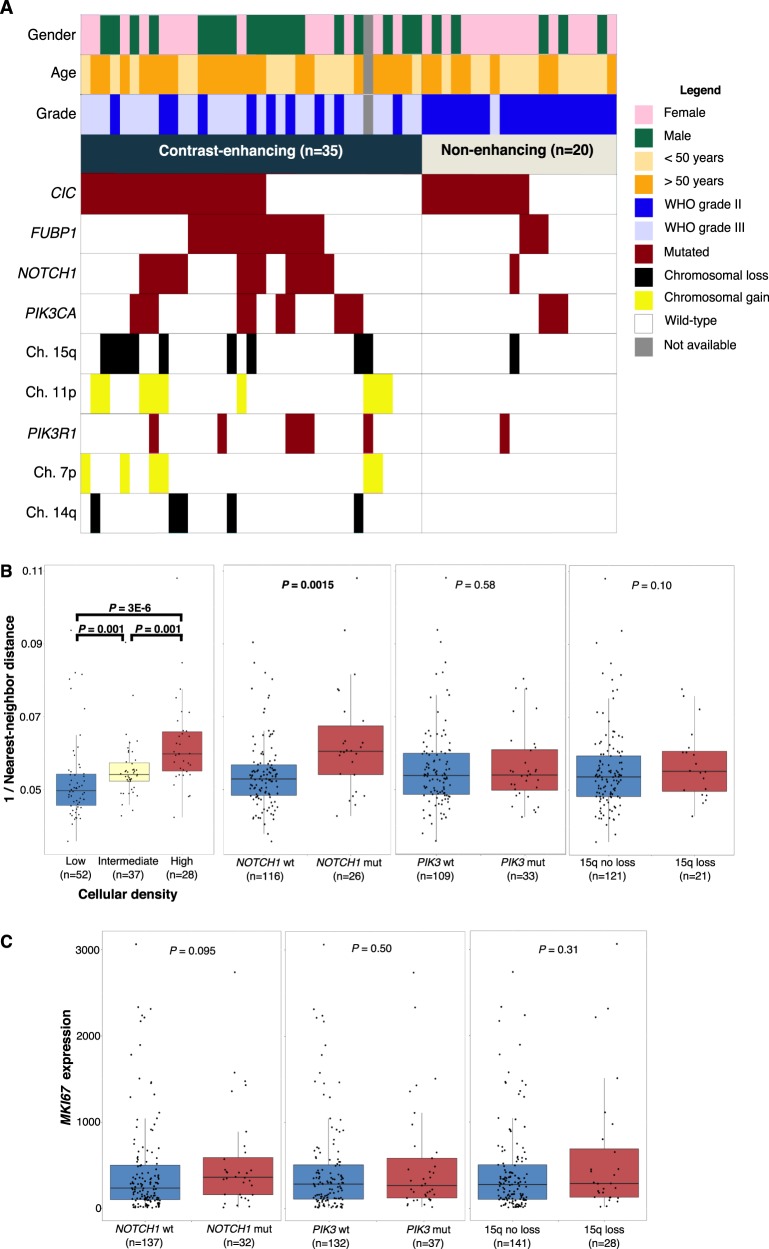
Genetic alterations associated with advanced disease progression **a** Waterfall plot illustrating the mutational landscape of oligodendrogliomas based on radiographic features of progression. **b** Boxplots demonstrating nearest-neighbor validation, and differential 1/nearest-neighbor distances in key genetic alterations of oligodendroglioma. **c** Boxplots for differential *MKI67* expression in key genetic alterations of oligodendroglioma. *P* values determined using Wilcoxon rank sum tests

Among 55 patients with MR imaging (Table S2), *NOTCH1* mutations were most strongly associated with CE+ tumors, with 13 of 14 *NOTCH1* mutants being CE+ (*P* = 0.008) (Fig. 3a). The combined *PI3K* group mutants were mostly CE+ (14 of 18; *P* = 0.054), and a similar trend was found among *FUBP1* mutants (14 of 17; *P* = 0.13). All 9 tumors with 11p gain were CE+ (*P* = 0.019). Although 5 of 5 tumors demonstrating loss of 14q were CE+, this did not reach statistical significance (*P* = 0.15). Similar trends were found with 15q loss (9 of 10 CE+; *P* = 0.075) and 7p gain (6 of 6 CE+; *P* = 0.076).

*NOTCH1* mutant oligodendrogliomas (*n* = 26) had higher cellular density than *NOTCH1* wild-type tumors (*n* = 126) and this difference was the most significant among all mutations and CNAs (*P* *=* 0.0015) (Fig. 3b). *FUBP1* mutants (*n* = 40) trended toward a higher cellular density compared to wild-type (*n* = 102; *P* *=* 0.10), and *CIC* (*n* = 88) and *PIK3* (*n* = 33) mutants did not show increased cell density (Fig. S3). Gains of 7p (*n* = 12) or 11p (*n* = 17) were significantly associated with higher cell densities (*P* *=* 0.006 and 0.03, respectively), and loss of 15q (*n* = 21) trended towards higher cellular density as well (*P* *=* 0.19) (Fig. 3b).

*NOTCH1* mutants (*n* = 32) had higher *MKI67* expression and this association was the strongest among all mutations and CNAs tested (*P* *=* 0.095) (Fig. 3c). *FUBP1*, *CIC*, and *PIK3* mutations were not strongly related to *MKI67* expression (Fig. S4). Although gain of 7p and 11p, and loss of 14q and 15q trended towards higher cellular proliferation, none reached statistical significance.

### Inactivation of the canonical Notch pathway is associated with disease progression measures

**Fig. 4:**
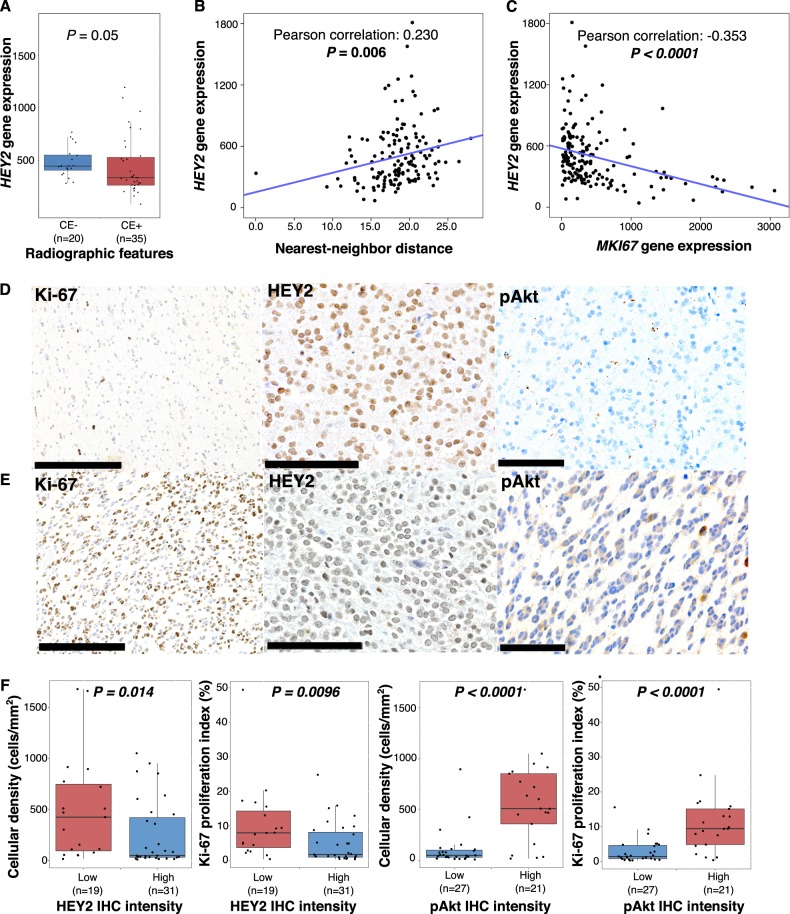
*HEY2* associations with advanced disease and validation cohort. **a** Boxplots demonstrating differential *HEY2* gene expression in CE− and CE+; *P* value determined using Wilcoxon rank-sum test. **b** Linear regression of *HEY2* gene expression and nearest-neighbor distance, demonstrating positive correlation. **c** Linear regression of *HEY2* and *MKI67* expression, demonstrating negative correlation. *P* values from Pearson correlation. **d** IHC showing high Ki-67 proliferation index (25%) (bar, 250 μm), with corresponding absent HEY2 expression (bar, 100 μm) and high pAkt expression (bar 100 μm). **e** IHC showing low Ki-67 proliferation index (1%) (bar, 250 μm), with corresponding high HEY2 expression (bar, 100 μm) and absent pAkt expression (bar, 100 μm). **f** HEY2 and pAKT IHC intensity as related to cellular density and Ki-67 proliferation indices

Since *NOTCH1* mutations were consistently and strongly associated with radiologic, pathologic, and molecular measures of progression, we investigated downstream targets of the canonical Notch pathway, including family members of hairy/enhancer of split 1 (*HES*) and hairy/enhancer of split with YRPW motif (*HEY*). Since nearly all (93%) *NOTCH1* mutations were located within the epidermal growth factor (EGF) like region, where they inhibit Notch activation, we hypothesized these targets would be down regulated in *NOTCH1* mutants.^17,18^ Expression of *HES1, HEY1*, and *HEY2* was reduced in CE+ tumors, with *HES1* and *HEY2* reaching statistical significance (*P* *=* 0.016 and 0.050, respectively) (Fig. 4a and Fig. S5). *HEY2* (Pearson correlation = 0.230, *P* *=* 0.006) was positively correlated with nearest-neighbor distance (Fig. 4b) and negatively correlated with cellular proliferation as approximated by *MKI67* expression (Pearson correlation = −0.353, *P* *<* 0.0001) (Fig. 4c). Negative correlations between *MKI67* expression and *HES1* (Pearson correlation = −0.152, *P* *=* 0.048) and *HEY1* (Pearson correlation = −0.082, *P* *=* 0.288) were also observed. Thus, among *HES* and *HEY* family members, *HES1*, *HEY1* and *HEY2* showed reduced expression with advanced disease, with *HEY2* showing the most consistent and statistically significant reductions.

### Alternate mechanisms of Notch pathway inactivation in oligodendroglioma

Recombinant signal binding protein for immunoglobulin kappa-J region (*RBPJ*), the nuclear binding partner of activated NOTCH1’s intracellular binding domain (NICD), was mutated (*n* = 5) or homodeleted (*n* = 1) in 3% (6 of 169) of oligodendrogliomas. *RBPJ* aberrations were mutually exclusive with *NOTCH1* mutations and were not present in *IDH* mutant or *IDH* wild-type astrocytomas. *RBPJ* altered tumors had greater *MKI67* expression compared to wild-type (*P* *=* 0.001) and showed a trend toward higher cell density (*P* *=* 0.20), but were not enriched in CE + tumors (Fig. S6A**)**. When *RBPJ* and *NOTCH1* mutant tumors were grouped (*n* = 38), *MKI67* expression and 1/nearest-neighbor distance showed stronger statistical significance in the combined group than in the group with *NOTCH1* mutants alone **(***P* *=* 0.0030 and 0.00039 for combined groups, respectively vs. *P* = 0.095 and 0.002 for *NOTCH1* mutants alone) (Fig. S6B). Thus, *RBPJ* mutation likely represents an alternative mechanism for Notch pathway inactivation in oligodendroglioma.

### Survival analysis reveals PIK3 mutations and reduced Notch target expression are associated with worse prognosis

A comprehensive analysis of clinical and genetic factors associated with survival was performed using a Cox proportional hazards models (Table 2 and Table S3). Univariable analysis revealed age and grade as strong predictors of poor OS (Hazards ratio (HR) 3.64 per 10 years, *P* *<* 0.0001; HR 6.61, *P* = 0.013, respectively). After adjusting for age and grade, the combination of *PIK3* mutations were found to confer poor prognosis (HR 3.11, *P* = 0.045). Among the downstream Notch target genes, increased *HES5* expression had a significant protective effect (HR 0.74, *P* *=* 0.024) after accounting for age and grade.

**Table 2:** Survival tables

Predictor	OS hazard ratio	*P-*value	Adjusted OS hazard ratio	*P*-value
^a^Age (per 10 yrs)	3.64	<0.0001	–	–
^a^Grade III (vs. II)	6.61	0.013	–	–
^a, c^*MKI67* exp.	1.58	0.0029	1.12	0.42
*NOTCH1* mut.	1.71	0.28	1.10	0.87
^b^*PIK3* mut.	1.97	0.15	3.11	0.045
*RBPJ* + *NOTCH1* mut.	1.81	0.210	0.85	0.76
^a^15q loss	3.52	0.007	1.48	0.47
^b, c^*HES5* exp.	0.82	0.086	0.74	0.024
^a, c^*HEY1* exp.	0.34	0.0009	0.86	0.72
^a, c^*HEY2* exp.	0.35	0.0001	0.79	0.54

	PFS hazard ratio	*P*-value	Adjusted PFS hazard ratio	*P*-value
Age (per 10 yrs)	1.12	0.28	–	–
^a^Grade III (vs. II)	2.24	0.046	–	–
^c^*MKI67* exp.	1.04	0.71	0.97	0.81
^a^*FUBP1* mut.	2.48	0.022	2.14	0.058
*NOTCH1* mut.	2.07	0.091	1.52	0.33
*PIK3* mut.	1.91	0.11	1.98	0.092
^a^ *RBPJ* + *NOTCH1 mut*.	2.47	0.021	1.86	0.13
^a, b^14q loss	3.70	0.010	3.90	0.0035
^a, b, c^*HEY1* exp.	0.41	0.0022	0.48	0.018

Cox proportional hazard models for overall survival (OS) and progression-free survival (PFS)

Multivariable OS adjusted for grade and age

Multivariable PFS adjusted for grade

*Mut* mutation

*Exp.* expression

^a^Significant on univariate analysis

^b^Significant after covariate adjustment

^c^Gene expression on a log2 scale, such that the hazard ratio is for each doubling of gene expression

Univariable analysis of PFS uncovered increased risk with grade III relative to grade II (HR 2.24, *P* = 0.046). *PIK3* (HR 1.98, *P* = 0.092) mutations trended toward increased risk of progression after accounting for tumor grade. Loss of 14q (HR 3.90 *P* *=* 0.0035) predicted more rapid time to progression after adjusting for grade. While *NOTCH1* mutants were not individually predictive of PFS, when combined with *RBPJ* altered tumors, the combined mutants predicted shorter time to first progression (HR 2.47, *P* *=* 0.021). After adjusting for grade, reduced *HEY1* (HR 0.48, *P* *=* 0.018) expression had a negative impact on PFS, while *HES5* trended in this direction (HR 0.86, *P* *=* 0.120). Complete survival analysis results in Table S3 and Fig. S7-S8.

### Translation and validation in clinical cases

We investigated 51 newly diagnosed cases of oligodendroglioma, grades II and III, from hospital archives. Pre-operative imaging was available for 47. We focused our IHC analysis on HEY2, since its gene expression showed greatest reduction in *NOTCH1* mutants, and pAkt, a downstream marker of PI3K activation (Figs. 4d, e).

Thirty-two tumors were WHO grade II and 19 were grade III; 21 tumors were CE− and 26 were CE+. By IHC analysis of HEY2, 20 tumors showed low expression and 31 showed high expression. Fourteen of 19 (73.7%) tumors with low HEY2 were CE+. Tumors with low HEY2 also had greater cell density (*P* = 0.014) and were more proliferative (*P* = 0.0096) than those with increased HEY2 staining (Fig. 4f). IHC investigation of pAkt found 27 tumors had low expression; 22 showed high expression; 15 of 20 (75%) tumors with pre-operative imaging and high pAkt expression were CE+. Tumors with high pAkt expression had greater cell density and were more proliferative (*P* *<* 0.0001, for both) (Fig. 4f).

## Discussion

We used a multi-faceted, technologically advanced, computational approach to identify molecular events associated with aggressive disease within molecularly defined oligodendroglioma (*IDH* mutant, 1p/19q co-deleted) and uncovered Notch pathway inactivation and PI3K activation as critical events. Our deep learning neural network methods analyzed multiplatform TCGA molecular data to generate protein-genetic and gene expression models of overall survival, and provided an objective ranking of clinical and molecular risk factors. In concordance with recent investigations,^8^ *NOTCH1* mutations were identified as one of the most highly weighted risk factors in our deep learning prognostic model, and was the genetic event most associated with disease progression in each endpoint assessed (MRI contrast-enhancement, cell density, and cellular proliferation). Therefore, inactivating point mutations of *NOTCH1* are one of the most clinically meaningful alterations in oligodendroglioma progression and might suggest that inactivation of the Notch pathway is more generally responsible for poor clinical outcomes.

The NOTCH family is an evolutionarily conserved set of transmembrane receptors that regulate numerous critical biological functions. Notch pathway is activated by extracellular ligand binding, followed by γ-secretase cleavage to release an active intracellular domain (NICD), which localizes to the nucleus and binds to its partner RBPJ to initiate transcription of downstream targets, including *HES* and *HEY* family members.^19,20^ Both activating and inactivating *NOTCH1* mutations have been described in cancer, including in oligodendroglioma.^8,20–23^ Inactivating mutations, such as those noted in oligodendroglioma and head and neck squamous cell carcinoma, are enriched within EGF-like regions and interfere with ligand-mediated pathway activation.^1,17,20,22,24–26^

Our results suggest inactivation of Notch signaling may be more relevant to oligodendroglioma progression than *NOTCH1* mutations alone. For example, reduced expression of Notch targets, namely *HES1*, *HEY1*, and especially *HEY2*, was seen in clinically progressed oligodendroglioma, while *HES5* expression was most associated with shorter survival on multivariable analysis. *HEY2* showed a strong positive correlation with cellular density and proliferation, beyond those of *NOTCH1* mutations alone, suggesting other Notch pathway members might be inactivated and lead to reduced downstream target activation.

Furthermore, we found mutations and deletions of *RBPJ*, the nuclear binding partner of NOTCH1 and a member of the canonical Notch pathway, are linked to advanced disease, providing additional evidence that Notch pathway inactivation may be a general progression mechanism. RBPJ normally recruits corepressor proteins and suppresses transcription of downstream targets, whereas active NOTCH1 binds RBPJ and initiates transcription.^27^ Genetic aberrations of *RBPJ* likely prevent active NOTCH1 from binding to the transcriptional complex. However, Notch-independent functions of RBPJ have also been described.^27^ *RBPJ* was mutated in 3% of our cohort and homozygously deleted in another case, which is relatively low, but consistent with other forms of cancer.^18,28^ Importantly, *RBPJ* alterations were mutually exclusive from *NOTCH1* mutations, showed strong trends of association with features of disease progression, and had reduced downstream target expression when considered independently. When cases with either *NOTCH1* mutations or *RBPJ* alterations were considered together, the combined group was more strongly associated with disease progression and pathway inactivation than either one alone, and was strongly associated with worse PFS, again raising the possibility that Notch pathway inactivation by multiple mechanisms may be associated with oligodendroglioma progression.

Other prognostically-significant chromosomal aberrations associated with disease progression uncovered by our analysis, including losses of 14q and 15q and gains of 7p, also harbor Notch pathway members, and may be mechanistically relevant to pathway inactivation and disease progression, but will require further investigation. Chromosome 14q contains genes that encode presenilin-1 (PSEN1), a component of the γ-secretase that activates Notch; NUMB, a Notch inhibitor; and jagged-2 (JAG2), a NOTCH receptor ligand. 15q, whose loss was nearly mutually exclusive with *NOTCH1* and *RBPJ* aberrations, contains genes coding for Delta-like 4 (DLL4), a NOTCH ligand; a disintegrin and metalloproteinase domain-containing protein 10 (ADAM10), a controller of NOTCH cleavage; and APH1B, a γ-secretase of NOTCH.^29^ Chromosome 7 contains the gene encoding lunatic fringe (LFNG), a key Notch signaling repressor, such that its overexpression could suppress Notch signaling.^29^ The identification of *RBPJ* mutations as a Notch pathway member associated with a poor prognosis, our link between gene expression of Notch pathway members to patient outcome, and the finding of downstream effectors of the Notch pathway, such as Hes and Hey family members, being downregulated in progressed oligodendrogliomas collectively point in the direction of uncovering other inactivating Notch family members, likely within amplified or deleted loci and providing a platform for assessing Notch pathway for predicting clinical behavior.

Mutations of *PIK3* subunits were highly weighted negative prognostic markers in our neural network analysis; were enriched in a subset of our endpoints of advanced disease; and were markers of shorter survival on multivariable analysis. Mutations of *PIK3CA* are activating, while those of *PIK3R* are inactivating, and both result in enhanced PI3K activity, with downstream activation of Akt and mammalian target of rapamycin, which are associated with aggressive clinical behavior in many cancers.^30^ Our neural network identified INPP4B, a known suppressor of PI3K signaling,^31^ as a protein whose increased expression was strongly associated with improved outcome. The PI3K pathway also strongly converges with SMAD2/3 and P38/MKK3 pathways, which were identified as among the most enriched with negative prognostic transcripts in our neural network.^15,16^ Lastly, our IHC analysis indicated pAkt expression was associated with higher-grade features and may have utility as a prognostic marker.

Importantly, our identification of Notch and PI3K pathways’ association with survival risk and disease progression does not demonstrate a causal or temporal relationship, and represents an inherent limitation of our study. The use of a machine-learning method does not resolve the issues of feature covariance that also limit the interpretation of models generated by more conventional approaches. We cannot prove *NOTCH1* or *PIK3* subunit mutations evolved temporally from a lower grade tumor, causing its progression. It is entirely possible oligodendrogliomas with Notch inactivation and PI3K activation are in fact distinct genetic subsets at their initiation and these tumors are more rapidly progressive. Longitudinal investigation of patient cohorts with primary and recurrent tumors is needed to identify temporal evolution.^32,33^ Future investigation will also require the elucidation of downstream targets of Notch and PI3K pathways that may drive glioma progression.

## Methods

### Study design

We used clinical and genomic data from the Open Access Data Tier of the TCGA LGG dataset for 169 oligodendroglioma (IDH mutant, 1p/19q co-deleted) (http://cancergenome.nih.gov/; last accessed September 7^th^, 2016). Clinical variables consisted of age, gender, extent of resection, overall survival time, survival status, progression-free survival time, and progression status; tumor characteristics included location and histologic grade based on the 2007 WHO brain tumor classification.^13^

### Deep learning survival model

We trained a Cox proportional hazards deep learning neural networks to model OS.^34^ Two models were constructed: (1) a genetic-protein model based on clinical factors (radiation therapy, histologic grade), age, gender, mutations, focal and arm-level copy number events (CNAs), and reverse phase protein array profiles, and 2) a transcriptional model based on mRNA sequencing factors alone. Mutations and CNAs were filtered using MutSig *P*-value threshold of 0.1, and Genomic Identification of Significant Targets in Cancer (GISTIC) *P*-value threshold of 0.25.^35,36^ The prognostic significance of each feature was assessed using mathematical derivatives to evaluate the sensitivity of risk to changes in feature values. Prognostic significance weights in the mRNA model were further used to perform pathway analysis to identify pathways enriched with either good or poor prognosis transcripts. Pathway analysis was performed with GSEA using the Canonical Pathways gene set from the MSigDB curated gene sets.

The accuracy of these modeling approaches in the oligodendroglioma cohort was evaluated using Monte-Carlo cross validation. We first randomly assigned 80% of samples to a training set, and the remaining 20% of samples to a testing set. A predictive model was trained using the training sample, and the accuracy of this model was evaluated using Harrell’s concordance-index (c-index) on the testing samples. This process was repeated for 20 randomized partitions of the dataset. For the genetic-proteomic model, a three layer network consisting of 100 neurons per layer was used. For the transcriptional model, a three layer network consisting of 500 neurons per layer was used. In both cases, these models were trained for 25 epochs using RMSprop optimization with a learning rate of 1e-3 and a dropout rate of 10%. Further details of this modeling approach are available in our previous work.^34^

Clinical data was obtained from the TCGA data portal (last accessed 22 January 2016). OS was defined as months from initial diagnosis to death. Survival curves were estimated using the Kaplan-Meier method; log-rank tests were used to compare curves between groups. Progression free survival (PFS) was defined as months from initial diagnosis to disease progression or death. PFS curves were estimated using the Kaplan–Meier method; log-rank tests were used to compare curves between groups. Single and multi-variable models (non machine-learning) were also fit using Cox regression under the proportional hazards assumption for OS and PFS.

### Genomic data

Gene expression, mutation, and CNA data were obtained from the TCGA portal (https://tcga-data.nci.nih.gov). Genetic alterations with at least 5% frequency were included in the analysis **(**Table S1A**)**. Variants were considered as mutants if there was an amino acid change and genes were filtered using *q* ≤ 0.05 in MutSig analysis. Mutations were then converted into dichotomous variables (mutation and wild-type). Arm level copy number data was obtained from GDAC GISTIC hosted analysis results (https://gdac.broadinstitute.org/). Values of chromosomal arm gain or loss were listed as a fraction of the chromosomal arm, where gains were positive values and losses were negative values. A threshold absolute value of 0.10 of the fraction of the chromosomal arm was used to signify chromosomal gain or loss. Frequency of chromosomal gains and losses are summarized in Table S1B.

### Radiographic imaging review

Preoperative MR imaging studies for TCGA patients were obtained from TCIA (http://www.cancerimagingarchive.net/; last accessed 8 February 2016) for 55 untreated patients. Institutional neuroradiologists and neurosurgeons reviewed MR images for the presence of unequivocal contrast-enhancement.

### Quantification of cellular density and nearest-neighbor analysis

Whole-slide digital pathology images (*n* = 142) were obtained from the CDSA (http://cancer.digitalslidearchive.net/; last accessed 11 August 2016). Images (20×) were analyzed using an image analysis algorithm to identify cell nuclei and to quantify cellular density in areas of tumor infiltration.^37^ The spacing between neighboring nuclei was calculated using KD-trees, and these distances were modeled using a Poisson point process. The densities of tumor and normal regions were deconvolved using a mixture Poisson model to identify the density parameter in tumor regions, *λ*_tumor_^−1^. The median tumor density across patients was used to define “less dense” and “more dense” categories. Cell density was also analyzed visually by a neuropathologist (JV), blinded to nearest neighbor analysis, and scored as: “low”, “intermediate”, or “high”. Algorithm and human assessments of density were highly concordant (Wilcoxon-rank sum < 0.05 level).

### Gene expression of MKI67 as a marker for cellular proliferation

A “high” category for *MKI67* expression was defined (≥700) to correspond to 15% MIB-1/Ki-67 labeling index using a linear regression model.^11^ Samples with *MKI67* < 700 were designated ‘low’.

### Statistics

Associations between contrast-enhancement and mutational status were calculated using the *χ*^2^ test for independence; for expected counts less than 5, Fisher’s exact test was used. Statistical associations between 2 groups of continuous or ordinal variables, such as the cellular density calls, were calculated using Wilcoxon rank-sum tests. The Pearson correlation coefficient was used to measure the linear dependence between continuous variables. All *P*-values reported are two-sided and regarded as statistically significant if *P* *<* 0.05. The software used for statistical analysis and graphical representations include: SPSS v23 (SPSS Statistics, IBM Corp., NY) and R Studio v0.99. All boxplots have the median marked as the center line, and whisker lines indicate the lower and upper quartiles (25 and 75%, respectively).

### Validation set

Fifty-one patients with primary oligodendroglioma (*IDH* mutant, 1p/19q co-deleted) were identified at Emory University Hospital with approval from the institution’s IRB committee and with a waiver of consent (IRB 00088647). MRIs were reviewed by a neuroradiologist (CAH) for contrast enhancement. Histologic slides were reviewed by two neuropathologists (DJB and JV). IHC staining was performed for Ki-67; a proliferation index was calculated using digital image analysis (Aperio Positive Pixel Count). Cell density was calculated by dividing cell count by area in regions of interest (mm^2^). IHC for Notch signaling was assessed using anti-HEY2 rabbit polyclonal antibody (catalog #AB5716, Millipore, 1:100) and for PI3K using anti-pAkt (S473) rabbit monoclonal antibody (#EP2109Y, Abcam, 1:100). HEY2 and pAkt IHC slides were reviewed and scored based on staining intensity. Selected samples underwent DNA isolation and focused sequencing of the NOTCH1 gene using Sanger sequencing, included the epidermal-growth-factor-like domain (EGF-like) spanning amino acids 300 to 500. Targeted sequencing was performed using a glioma gene panel on the Fluidigm platform.

## Electronic supplementary material

Supplemental Figures and tables

## Data Availability

All data used in this investigation is accessible in Supplementary Data File S1.

## Electronic supplementary material

**Supplementary information** accompanies the paper on the *npj Precision Oncology* website (10.1038/s41698-018-0067-9).
