# Supplementary material for: Multi-faceted computational assessment of risk and progression in oligodendroglioma implicates NOTCH and PI3K pathways
Source: NPJ Precis Oncol. 2018 Nov 6;2:24. doi: 10.1038/s41698-018-0067-9 (PMC6219505; doi:10.1038/s41698-018-0067-9)
Supplement: Supplementary file 1 — Supplemental Figures and tables [file 41698_2018_67_MOESM1_ESM.pdf]

Table S1A. Gene names and frequency of mutations for oligodendroglioma in the TCGA database.

Gene Name	Frequency of Mutations
**IDH1**	154
**CIC**	104
**FUBP1**	45
**PI3K comb.**	37
**NOTCH1**	32
**PIK3CA**	26
**ZBTB20**	16
**IDH2**	15
**PIK3R1**	12
**ARID1A**	11
**NF1**	8
**SMARCA4**	7
**TP53**	7
**CREBZF**	6
**TCF12**	6
**ANKRD30A**	5
**RBPJ**	5
**ATRX**	4
**MYH4**	4
**SOX4**	4
**CDKN2C**	3
**EGFR**	3
**EMG1**	3
**MYH8**	3
**SLC6A3**	3
**ARL6**	2
**CXorf22**	2
**KEL**	2
**KPRP**	2
**KRT15**	2
**LHFPL1**	2
**NRAS**	2
**OR4S2**	2
**OR5D18**	2
**PDGFRA**	2
**SEMA3E**	2
**SPANXD**	2
**TRPA1**	2
**UGT2A3**	2
**VSIG4**	2
**ZNF709**	2
**CDH18**	1
**DCAF12L2**	1
**KRAS**	1
**LZTR1**	1
**MED9**	1
**MGAT4C**	1
**OPRK1**	1
**OR2A12**	1
**OR2A25**	1
**OR4P4**	1
**OR8K3**	1
**OR9G1**	1
**P2RY11**	1
**PTEN**	1
**PTPN11**	1
**RAP2C**	1
**RB1**	1
**RPL5**	1
**SPTA1**	1
**SSTR4**	1
**ST3GAL6**	1
**STK19**	1
**TEAD3**	1
**TREML2**	1
**TRIM58**	1
**TYRP1**	1
**ZPBP**	1

Supplemental Table 1B. Gain and loss of whole chromosome arms of oligodendroglioma within the TCGA database.

Chromosome	Loss	Gain
**1p**	166	0
**1q**	12	1
**4p**	45	0
**4q**	50	0
**7p**	0	16
**7q**	0	24
**9p**	21	2
**9q**	15	3
**11p**	0	19
**11q**	0	24
**12p**	5	4
**13q**	28	0
**14q**	20	1
**15q**	27	0
**17p**	2	7
**17q**	1	8
**18p**	31	0
**18q**	33	0
**19p**	7	57
**19q**	167	0
**21q**	2	9
**22q**	4	17
**Xp**	33	0
**Xq**	32	1

Table S2. Patient demographics. Clinical characteristics of patients with pre-operative radiographic imaging from The Cancer Genome Atlas database, with confirmed diagnosis of oligodendroglioma (i.e. IDH-mutant, 1p19q co-deleted glioma).

**Characteristic**	**Total (N=55)**
Histological subtype – no. (%)	
Oligodendroglioma	
Grade II	23 (41.8)
Grade III	24 (43.6)
Oligoastrocytoma	
Grade II	5 (9.1)
Grade III	1 (1.8)
Astrocytoma	
Grade II	1 (1.8)
Grade III	0 (0.0)
Age at diagnosis (yrs)	
Mean	49.4 $\pm$ 13.2
Range	20–75
Radiographic features present – no./total no. (%)	
- Contrast-enhanced	20/55 (36.4)
+ Contrast-enhanced	35/55 (63.6)
Tumor location – no./total no. (%)	
Frontal lobe	40/55 (72.7)
Parietal lobe	7/55 (12.7)
Temporal lobe	3/55 (5.5)
Other	5/55 (9.1)

Table S3. Complete survival tables. Cox proportional hazard models for overall survival (OS) and progression-free survival (PFS); multivariate OS adjusted for grade and age; multivariate PFS adjusted for grade. GTR: gross total resection; mut: mutation; exp.: expression; *Significant on univariate analysis; †significant on multivariate analysis; ‡gene expression on a log2 scale, such that the hazard ratio is for each doubling of gene expression.

Predictor	OS Hazard Ratio (95% conf. interval)	*P*-value	Adjusted OS Hazard Ratio (95% conf. interval)	*P*-value
***Age (per 10 yrs)**	**3.64 (2.16-6.11)**	**<0.0001**	—	—
Histologic astrocytoma vs. oligodendroglioma	2.94 (0.046-2.70)	0.31	—	—
Extent of resection (GTR vs. less than GTR)	0.65 (0.24-1.68)	0.37	—	—
***Grade III (vs. II)**	**6.61 (2.08-20.95)**	**0.013**	—	—
***‡*MKI67* exp.**	**1.58 (1.17-2.14)**	**0.0029**	1.12 (0.84-1.50)	0.42
ATRX mut.	NA	NA	<0.0001	0.998
CIC mut.	0.65 (0.27-1.55)	0.33	0.44 (0.17-1.15)	0.095
FUBP1 mut.	1.70 (0.63-4.53)	0.31	2.60 (0.84-8.00)	0.096
NOTCH1 mut.	1.71 (0.65-4.50)	0.28	1.10 (0.37-3.27)	0.87
**†*PIK3* mut.**	1.97 (0.78-4.97)	0.15	**3.11 (1.02-9.47)**	**0.045**
RBPJ + *NOTCH1* mut.	1.81 (0.71-4.61)	0.210	0.85 (0.30-2.40)	0.76
TP53 mut.	1.36 (0.18-10.33)	0.77	1.16 (0.14-9.65)	0.893
7p gain	1.90 (0.42-8.52)	0.44	1.23 (0.26-5.68)	0.795
11p gain	2.08 (0.58-7.48)	0.26	0.60 (0.16-2.33)	0.463
14q loss	3.04 (0.94-9.77)	0.063	1.49 (0.39-5.62)	0.559
***15q loss**	**3.52 (1.41-8.82)**	**0.007**	1.48 (0.51-4.30)	0.47
‡*HES1* exp.	0.60 (0.35-1.05)	0.071	0.86 (0.47-1.56)	0.611
‡*HES2* exp.	1.01 (0.93-1.10)	0.76	1.00 (0.89-1.12)	0.954
**†‡*HES5* exp.**	0.82 (0.65-1.03)	0.086	**0.74 (0.57-0.96)**	**0.024**
***‡*HEY1* exp.**	**0.34 (0.18-0.64)**	**0.0009**	0.86 (0.38-1.95)	0.72
***‡*HEY2* exp.**	**0.35 (0.21-0.60)**	**0.0001**	0.79 (0.37-1.68)	0.54

	PFS Hazard Ratio (95% conf. interval)	*P*-value	Adjusted PFS Hazard Ratio (95% conf. interval)	*P*-value
Age (per 10 yrs)	1.12 (0.86-1.66)	0.28	—	—
***Histologic astrocytoma vs. oligodendroglioma**	**4.77 (1.09-20.78)**	**0.038**	—	—
Extent of resection (GTR vs. less than GTR)	0.66 (0.30-1.44)	0.30	—	—
***Grade III (vs. II)**	**2.24 (1.01-4.94)**	**0.046**	—	—
‡*MKI67* exp.	1.04 (0.84-1.29)	0.71	0.97 (0.78-1.21)	0.81
ATRX mut.	2.93 (0.38-22.67)	0.30	2.09 (0.27-16.3)	0.483
CIC mut.	0.61 (0.29-1.27)	0.18	0.58 (0.28-1.20)	0.140
******FUBP1*** **mut.**	**2.48 (1.14-5.38)**	**0.022**	2.14 (0.98-4.69)	0.058
NOTCH1 mut.	2.07 (0.93-4.60)	0.091	1.52 (0.66-3.53)	0.33
PIK3 mut.	1.91 (0.87-4.23)	0.11	1.98 (0.89-4.40)	0.092
****RBPJ + NOTCH1 mut.***	**2.47 (1.14-5.34)**	**0.021**	1.86 (0.82-4.20)	0.13
TP53 mut.	2.57 (0.60-11.03)	0.21	2.57 (0.59-11.26)	0.211
7p gain	0.55 (0.07-4.11)	0.56	0.42 (0.06-3.19)	0.404
11p gain	1.31 (0.38-4.44)	0.67	1.19 (0.35-4.08)	0.779
***†14q loss**	**3.70 (1.49-9.20)**	**0.010**	**3.90 (1.56-9.74)**	**0.0035**
15q loss	1.75 (.073-4.19)	0.21	1.72 (0.70-4.22)	0.239
‡*HES1* exp.	0.75 (0.49-1.15)	0.18	0.70 (0.45-1.10)	0.118
‡*HES2* exp.	1.01 (0.96-1.08)	0.67	1.02 (0.95-1.10)	0.638
†‡*HES5* exp.	0.83 (0.68-1.02)	0.076	0.86 (0.71-1.04)	0.120
†‡**HEY1*** **exp.**	**0.41 (0.23-0.72)**	**0.0022**	**0.475 (0.26-0.88)**	**0.018**
‡*HEY2* exp.	0.87 (0.53-1.44)	0.59	0.96 (0.57-1.61)	0.869

Supplemental Figures

Figure S1. Boxplots of the Monte-Carlo cross validation of the genetic-protein and gene expression survival neural networks. The median c-index of the tested genetic-protein models was 0.8 (+/- 0.124), while the median c-index of the tested gene expression models was 0.752 (+/- 0.196).

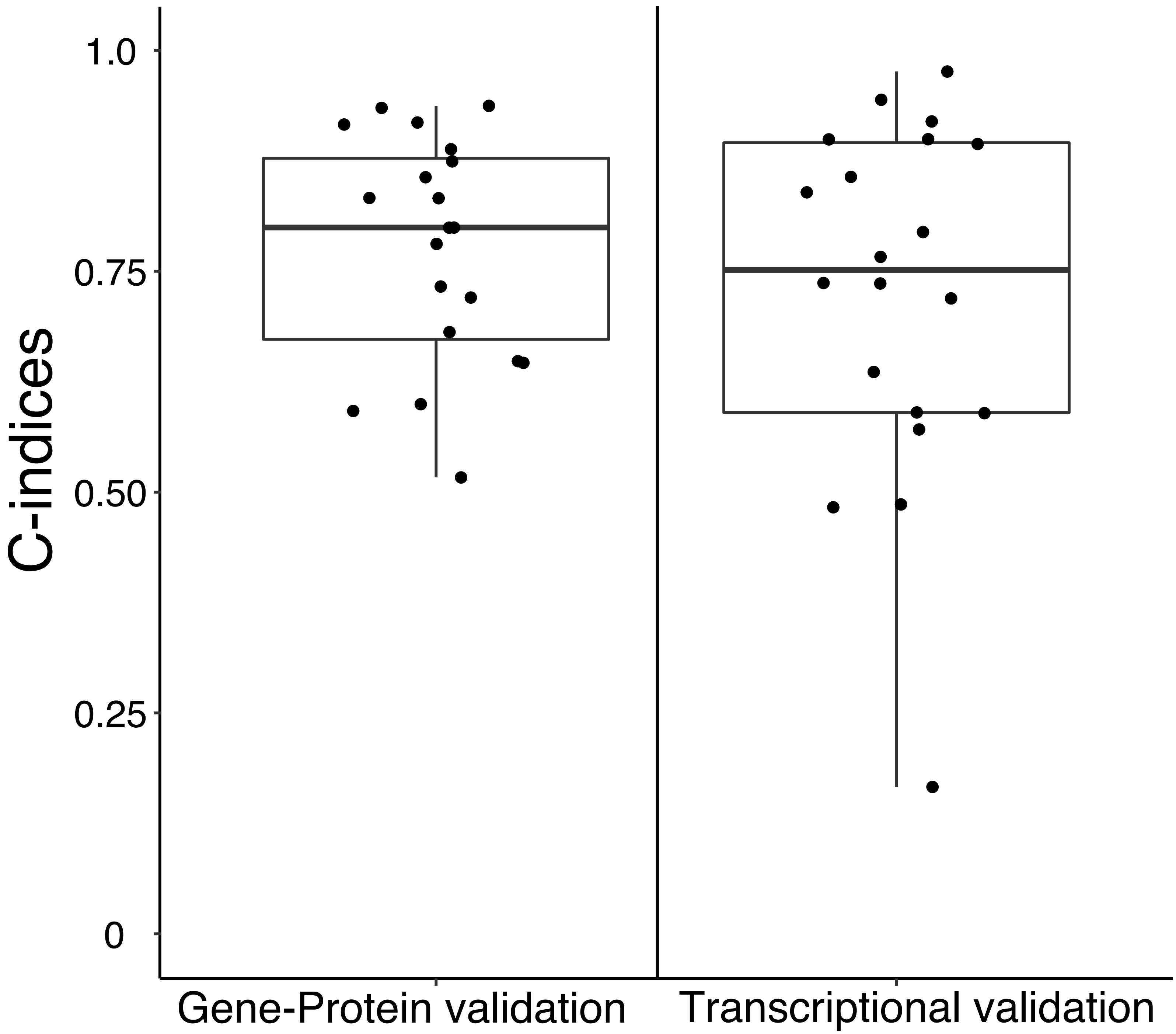

C-indices
1.0
0.75
0.50
0.25
0
Gene-Protein validation
Transcriptional validation

Figure S2. A waterfall plot of the most frequent genetic alterations found in oligodendroglioma.

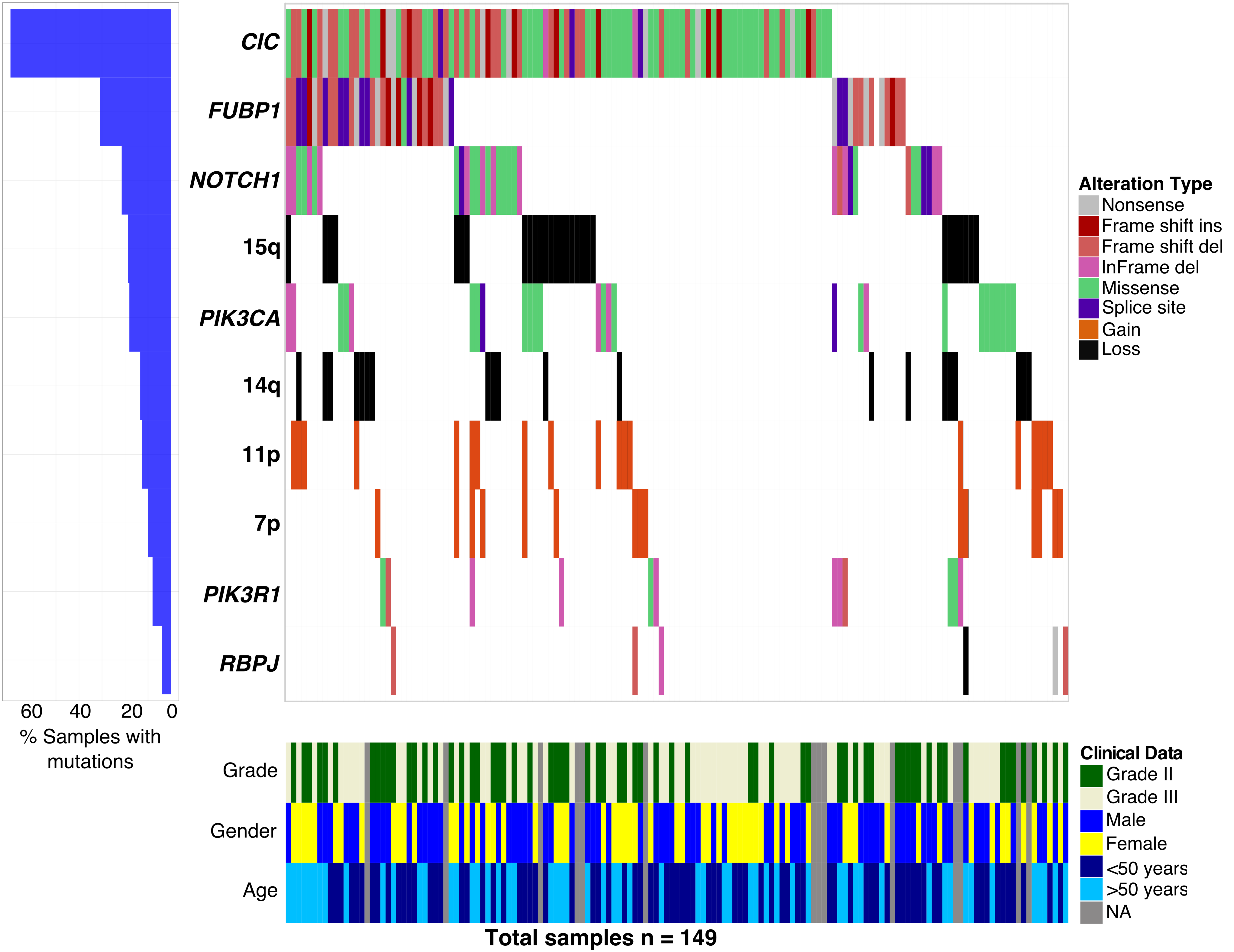

CIC
FUBP1
NOTCH1
15q
PIK3CA
14q
11p
7p
PIK3R1
RBPJ
60
40
20
0
% Samples with mutations
Alteration Type
Nonsense
Frame shift ins
Frame shift del
InFrame del
Missense
Splice site
Gain
Loss
Grade
Gender
Age
Clinical Data
Grade II
Grade III
Male
Female
<50 years
>50 years
NA
Total samples n = 149

Figure S3. Boxplots demonstrating the differential cellular density using nearest neighbor distances between altered and unaltered oligodendroglioma.

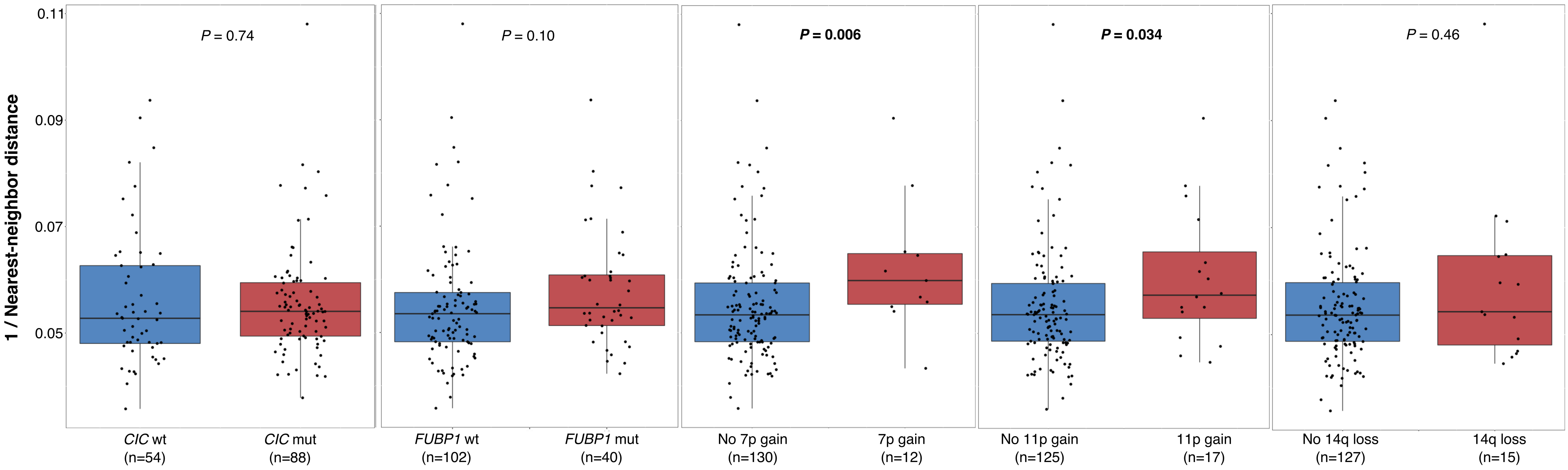
1 / Nearest-neighbor distance
0.11
0.09
0.07
0.05
P = 0.74
P = 0.10
P = 0.006
P = 0.034
P = 0.46
CIC wt (n=54)
CIC mut (n=88)
FUBP1 wt (n=102)
FUBP1 mut (n=40)
No 7p gain (n=130)
7p gain (n=12)
No 11p gain (n=125)
11p gain (n=17)
No 14q loss (n=127)
14q loss (n=15)

Figure S4. Boxplots demonstrating differences in *MKI67* gene expression, and thus cellular proliferation, between altered and unaltered oligodendroglioma.

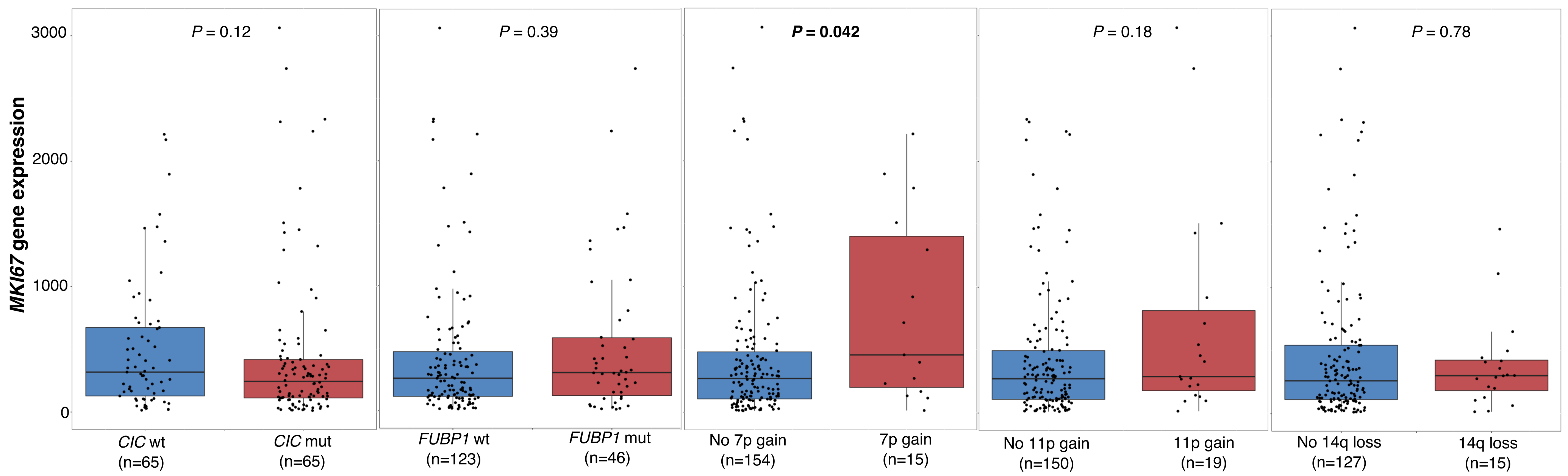
MKI67 gene expression
3000
2000
1000
0
P = 0.12
P = 0.39
P = 0.042
P = 0.18
P = 0.78
CIC wt (n=65)
CIC mut (n=65)
FUBP1 wt (n=123)
FUBP1 mut (n=46)
No 7p gain (n=154)
7p gain (n=15)
No 11p gain (n=150)
11p gain (n=19)
No 14q loss (n=127)
14q loss (n=15)

Figure S5. Boxplots illustrating differences in gene expression of downstream Notch pathway targets, HES1 and HEY1, between contrast enhancing and non-enhancing oligodendroglioma.

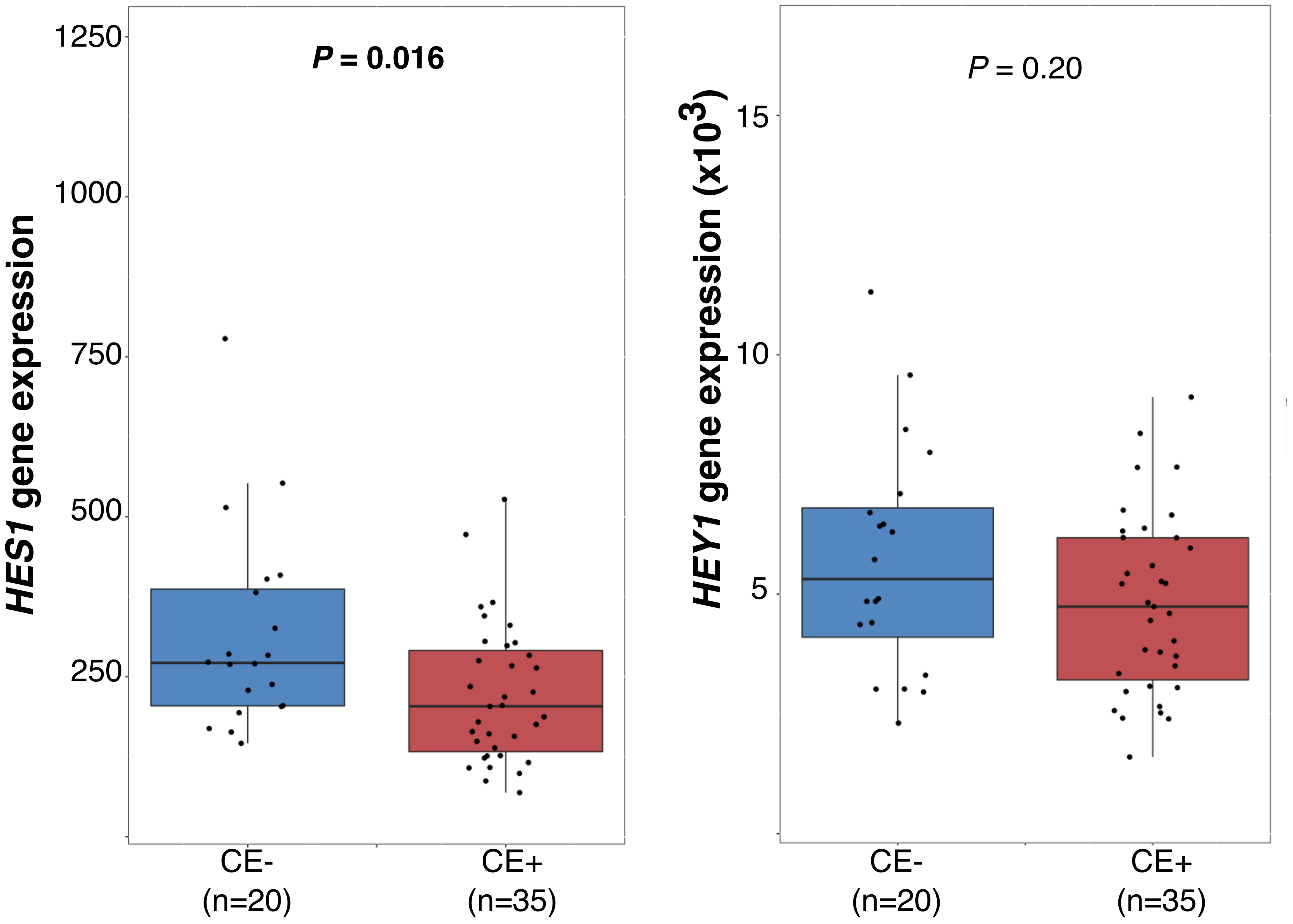

HES1 gene expression
1250
1000
750
500
250
P = 0.016
CE-
(n=20)
CE+
(n=35)
HEY1 gene expression (x10^3)
15
10
5
P = 0.20
CE-
(n=20)
CE+
(n=35)

Figure S6. Boxplots demonstrating the differences in cellular density and proliferation in tumors with and without RBPJ mutations, and those tumors with both NOTCH1 and RBPJ mutations and without.

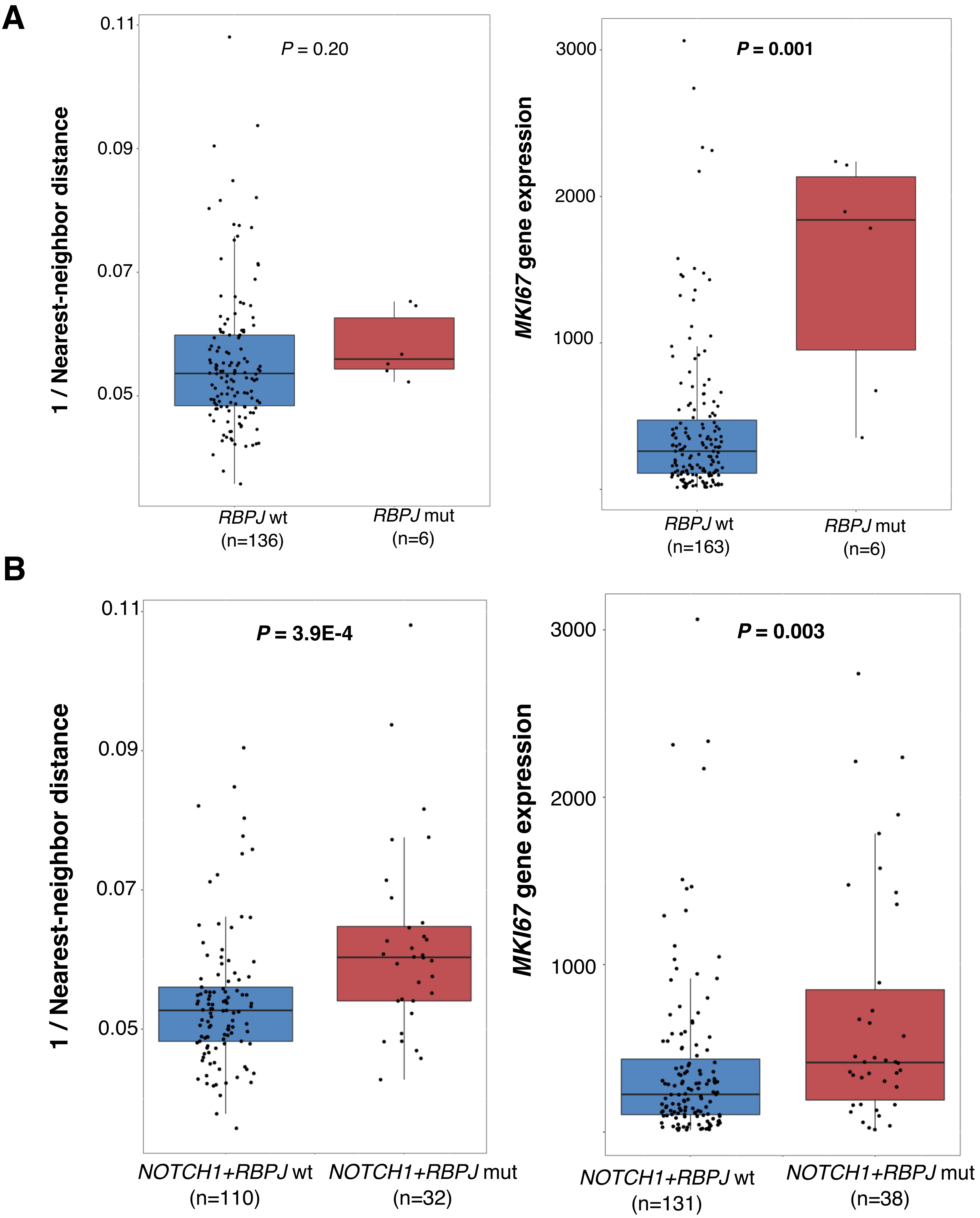

A
1 / Nearest-neighbor distance
0.11
0.09
0.07
0.05
P = 0.20
RBPJ wt
(n=136)
RBPJ mut
(n=6)
MKI67 gene expression
3000
2000
1000
P = 0.001
RBPJ wt
(n=163)
RBPJ mut
(n=6)
B
1 / Nearest-neighbor distance
0.11
0.09
0.07
0.05
P = 3.9E-4
NOTCH1+RBPJ wt
(n=110)
NOTCH1+RBPJ mut
(n=32)
MKI67 gene expression
3000
2000
1000
P = 0.003
NOTCH1+RBPJ wt
(n=131)
NOTCH1+RBPJ mut
(n=38)

Figure S7. Kaplan-Meier plots for overall survival (OS) of the key genetic alterations found in oligodendroglioma. Statistical significance was assessed using log-rank tests.

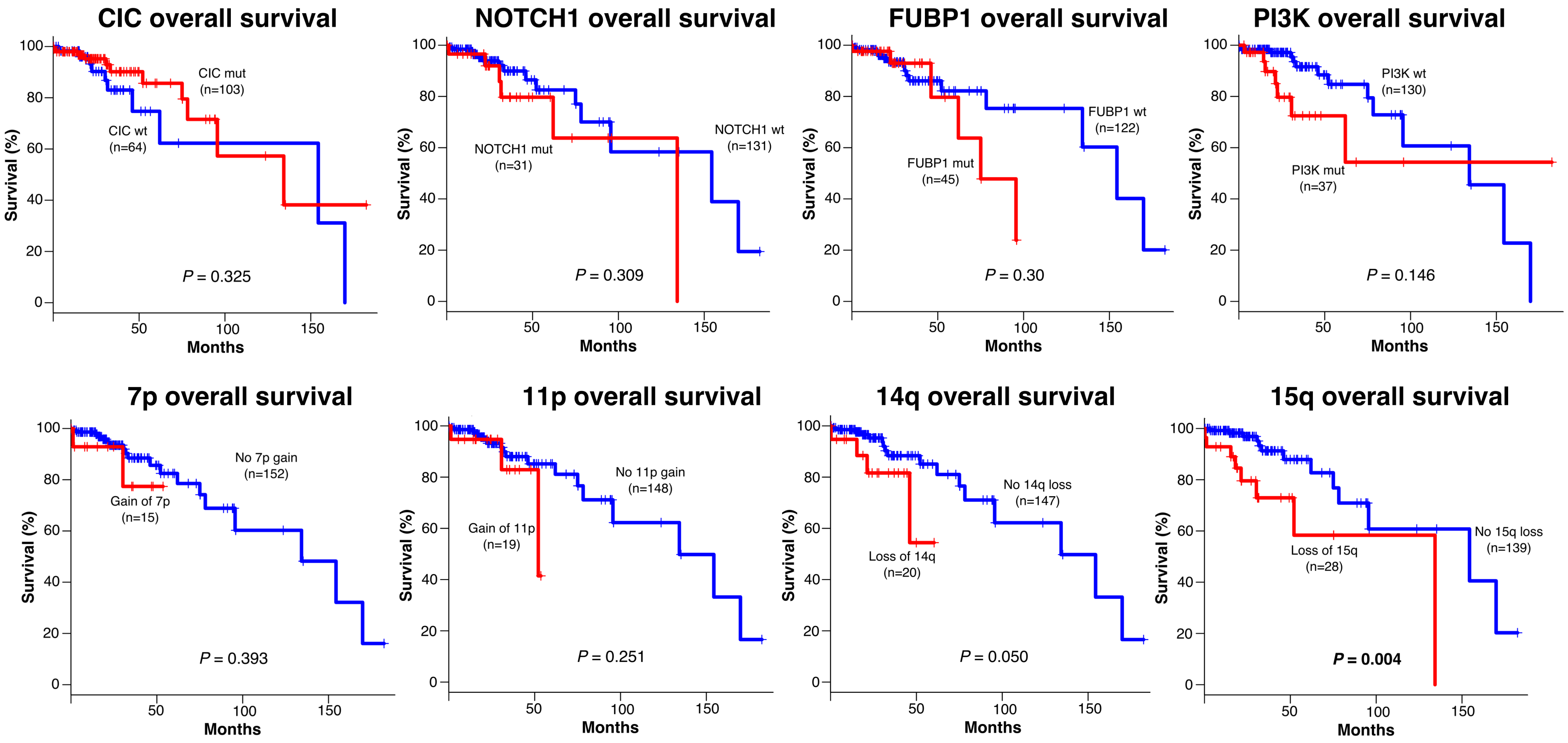
CIC overall survival
CIC mut
(n=103)
CIC wt
(n=64)
P = 0.325
Survival (%)
Months
NOTCH1 overall survival
NOTCH1 wt
(n=131)
NOTCH1 mut
(n=31)
P = 0.309
Survival (%)
Months
FUBP1 overall survival
FUBP1 wt
(n=122)
FUBP1 mut
(n=45)
P = 0.30
Survival (%)
Months
PI3K overall survival
PI3K wt
(n=130)
PI3K mut
(n=37)
P = 0.146
Survival (%)
Months
7p overall survival
No 7p gain
(n=152)
Gain of 7p
(n=15)
P = 0.393
Survival (%)
Months
11p overall survival
No 11p gain
(n=148)
Gain of 11p
(n=19)
P = 0.251
Survival (%)
Months
14q overall survival
No 14q loss
(n=147)
Loss of 14q
(n=20)
P = 0.050
Survival (%)
Months
15q overall survival
No 15q loss
(n=139)
Loss of 15q
(n=28)
P = 0.004
Survival (%)
Months

Figure S8. Kaplan-Meier plots for progression-free survival (PFS) of the key genetic alterations found in oligodendroglioma. Statistical significance was assessed using log-rank tests.

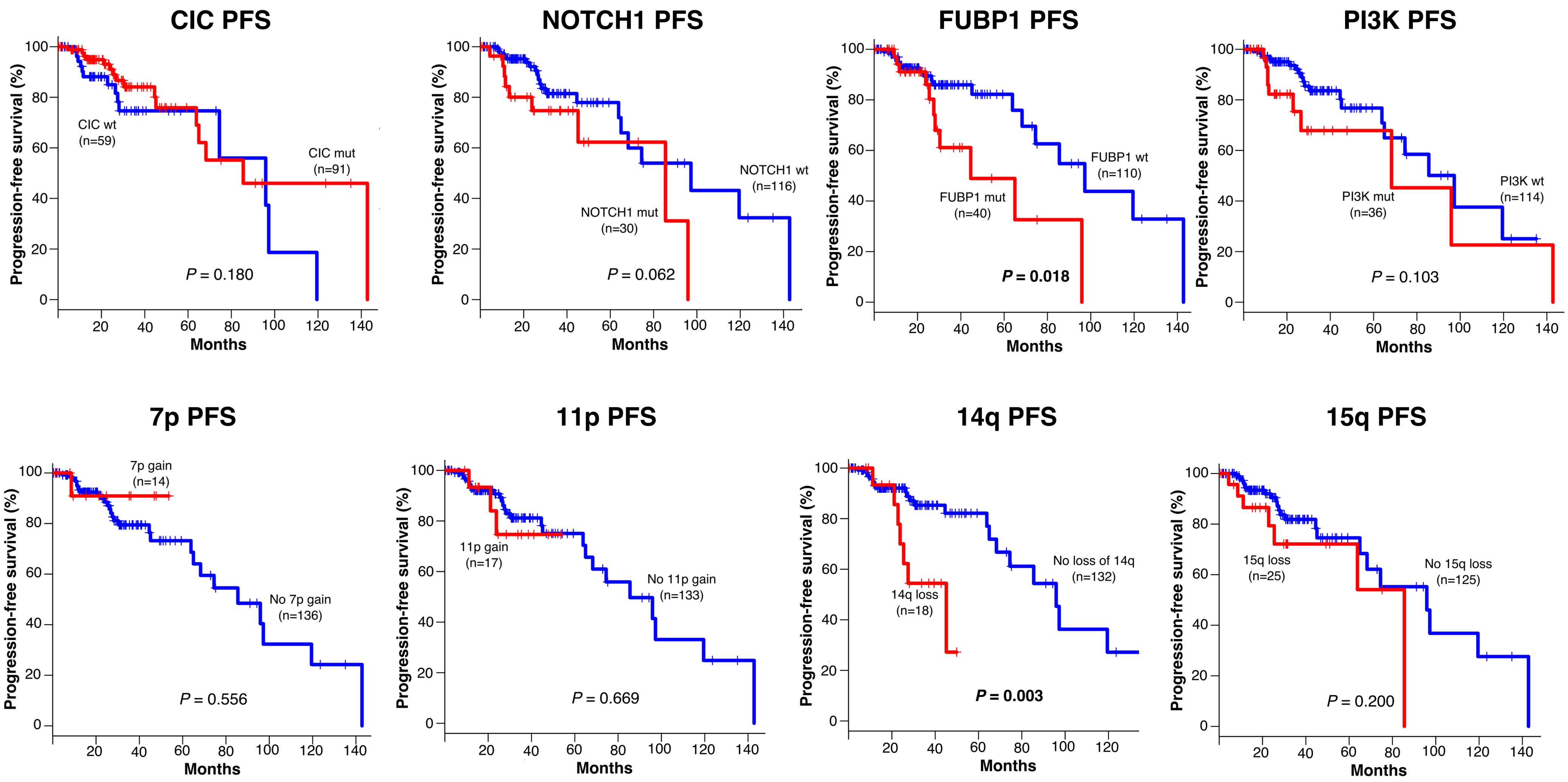
CIC PFS
CIC wt
(n=59)
CIC mut
(n=91)
P = 0.180
NOTCH1 PFS
NOTCH1 wt
(n=116)
NOTCH1 mut
(n=30)
P = 0.062
FUBP1 PFS
FUBP1 wt
(n=110)
FUBP1 mut
(n=40)
P = 0.018
PI3K PFS
PI3K wt
(n=114)
PI3K mut
(n=36)
P = 0.103
7p PFS
7p gain
(n=14)
No 7p gain
(n=136)
P = 0.556
11p PFS
11p gain
(n=17)
No 11p gain
(n=133)
P = 0.669
14q PFS
No loss of 14q
(n=132)
14q loss
(n=18)
P = 0.003
15q PFS
15q loss
(n=25)
No 15q loss
(n=125)
P = 0.200
Progression-free survival (%)
Months
